# Identification and Genome Characterization of a Novel Muscovy Duck-Origin Goose Parvovirus with Three Recombinant Regions between Muscovy Duck Parvovirus and Goose Parvovirus

**DOI:** 10.1155/2024/1018317

**Published:** 2024-03-06

**Authors:** Hongwei Liu, Zhuoran Xu, Shao Wang, Xiaoxia Cheng, Shifeng Xiao, Xiaoli Zhu, Min Zheng, Fengqiang Lin, Hui Dong, Dandan Jiang, Shaoying Chen, Shilong Chen

**Affiliations:** ^1^Institute of Animal Husbandry and Veterinary Medicine, Fujian Academy of Agriculture Sciences, Fuzhou 350003, China; ^2^College of Animal Sciences, Fujian Agriculture and Forestry University, Fuzhou 350002, China; ^3^Fujian Animal Diseases Control Technology Development Center, Fuzhou 350003, China

## Abstract

Muscovy duck-origin goose parvovirus (MDGPV) is a new virus resulting from the natural recombination of Muscovy duck parvovirus (MDPV) and goose parvovirus (GPV). Previously identified MDGPV strains were found to have two recombination regions, one in the P9 promoter to the NS region and one in the VP3 gene, or only one recombination in the VP3 gene. In 2022, a novel strain of MDGPV known as 2022FZ was identified from China's mainland. Complete genome sequence analysis showed that there were three recombination regions in this strain: one located in the P9 promoter-NS (425–612 nt) region, one in the NS2 (1,483–1,824 nt) region, and one in the VP3 (3,124–4,248 nt) region, respectively. The recombination regions in the P9 promoter-NS, NS2, and VP3 genes were substituted with the relevant GPVs sequences, whereas the MDPV virulent strain served as the skeleton in this instance. In addition, the 2022FZ strain had multiple unique aa mutations in the NS protein and the VP protein. The Muscovy duckling challenge test showed that MDGPV-2022FZ is less pathogenic to Muscovy ducklings than two recombinant or sole recombinant MDGPV strains. For the first time, our study identified a three-region recombinant MDGPV strain and detected the novel recombination event in the NS2 gene. These results contribute to our understanding of the pathogenicity and genetic diversity of duck parvovirus.

## 1. Introduction

Waterfowl parvoviruses (*Parvoviridae*: *Dependovirus*) are divided into two major groups according to host susceptibility and complete genomic features [1], including goose parvoviruses (GPVs) and Muscovy duck parvoviruses (MDPVs) [1–3]. The MDPV is only pathogenic to Muscovy ducklings, causing a 3-week-disease [3], while GPV causes Derzy's disease in gooselings and Muscovy ducklings [4, 5] and short beak and dwarfism syndrome in Peking ducks and mule ducks [6–8]. The genomes of waterfowl parvoviruses are around 5.1 kb long and comprise a single stranded, negative sense, encased DNA strand with two open reading frames (ORFs) [9]. The left ORF includes two nonstructural genes (NS1 and NS2) and encodes the replication protein1 (Rep1) and Rep2, respectively [9]. The Rep2 protein is derived from Rep1 and is shorter in its N-terminal domain. The correct ORF encodes three capsid proteins (VP1, VP2, and VP3). By selectively cleaving mRNA and using distinct start codons, these three proteins come together to form the viral capsid in a ratio of 1 : 1 : 8 [1, 9, 10].

GPVs and MDPVs have similar gene structures and patterns of gene expression, whereby genetic recombination is prone to be generated after the simultaneous infection of the same host by two different parvoviruses. Genetic recombination plays an important role in waterfowl parvovirus evolution, and the past two decades have yielded evidence of the natural recombination between MDPV and GPV resulting in recombinant waterfowl parvovirus [11–16]. Based on the serological investigation and phylogenetic analysis of the VP3 gene, these recombinant waterfowl parvoviruses are closely related to GPV and are designated Muscovy duck-origin GPV (MDGPV) [15, 17–19]. The MDGPV strain PT (MDGPV/PT), a sole recombination waterfowl parvovirus, was the first recombinant strain between MDPV and GPV isolated in 1997, exhibiting a 1.1 kb region in the middle of the VP3 gene replaced with the homologous region of GPV, using the classical MDPV genome as the backbone [15, 18]. Since 2006, MDGPV strains have been identified with two genetic recombination events in their P9 promoter region and 1.1 kb region in the middle of the VP3 gene [12]. An MDGPV infection has a higher death rate than the traditional 3-week illness, but it shares the intestinal embolism diagnostic characteristic seen in Derzy's disease [12, 18, 20].

Both single- and double-recombinant MDGPV strains are highly pathogenic to Muscovy ducklings, leading to immense economic loss in the Muscovy duck industry in China [12, 18]. In this molecular epidemiological study, a novel three-region recombinant MDGPV strain was identified for the first time in Mainland China.

## 2. Materials and Methods

### 2.1. Virus Strain and Viral Propagation

The MDGPV/2022FZ strain was isolated from dead Muscovy ducklings suspected of MDGPV infection from a farm in Fuzhou, Fujian province, China, as described previously [6]. The isolated MDGPV/2022FZ strain was inoculated into the allantoic cavity of 11-day-old embryonated Muscovy duck eggs. The allantoic fluid of embryos that died was pooled and the viral titer was determined by latex agglutination (LA) assay, as described previously [6].

### 2.2. Viral DNA Extraction, Genome Amplification, and Sequencing

Viral DNA was extracted from the infected allantoic fluid using a HiPure Viral RNA/DNA Kit (Vazyme, Nanjing, China), according to the manufacturer's instructions. Seven overlapping DNA fragments, covering the entire viral genome, were amplified by PCR using primer pairs designed from the GenBank-derived genomic sequence of the MDGPV/PT strain (KY511293) (Table 1). All PCR products were purified using the DNA gel extraction kit (Tsingke, Beijing, China) and then ligated into the TA/Blunt Zero Cloning vector (Vazyme, Nanjing, China). At least three positive clones were randomly selected from each case and sequenced by Sangon Biotech Shanghai Co., Ltd.

### 2.3. Sequence Comparison, Recombination Analysis, and Phylogenetic Tree Construction

Complete genomic sequences of waterfowl parvovirus strains were retrieved from GenBank, including the Muscovy duck-origin GPV strains (PT, ZW, NM100, SAAS-SHNH, and GD201911), classical MDPV strains (P, YY, FM, FZ91-30, and FJM5), and GPV strains (B, 82-0321, YZ99-6, DY16, LH, and SYG61v) (Table 2). The Recombination Detection Program SimPlot v3.5.1 and Recombination Detection Program v.4.100 (RDP4) were used to detect possible recombination events. To reveal the evolutionary relationships between the 2022FZ strain and other waterfowl parvovirus strains, phylogenetic trees were constructed in MEGA 11 using the whole genomic sequence and the NS2 and VP3 recombinant regions, respectively. Branch support was assessed through 1,000 bootstrap replicates.

### 2.4. Ducklings Challenge Experiment

To evaluate the pathogenicity of the 2022FZ recombinant strain, 40 6-day-old Muscovy ducklings were randomly divided into four groups, with ten ducklings per group. The ducklings in Group 1 were injected intramuscularly in a leg with 0.6 ml of strain 2022FZ (5^th^-passage allantoic fluid virus with 2^5.0^ LA titer). The ducklings in Groups 2 and 3 were injected subcutaneously with the same dose of MDGPV/PT (single-region recombinant MDGPV) or MDGPV/ZZ (two-region recombinant MDGPV) as the positive control, while the ducklings in Group 4 were injected with 0.6 ml of sterile phosphate-buffered saline as a mock control. The Muscovy ducklings in each group were reared in isolators, and any clinical signs were monitored daily for a total of 14 days.

### 2.5. Detection of GPV and MDPV Antigens by IFA

Our previous studies have demonstrated that MDGPV is a GPV-like isolate through serological testing [15]. To verify the serotype of MDGPV/2022FZ, the heart and liver samples of dead ducks after MDGPV/2022FZ infection were detected GPV and MDPV antigens by indirect immunofluorescence assay (IFA) as the method described previously [6]. The specific GPV Mab G-E16 and MDPV Mab M-f21 were prepared in our laboratory. Fluorescein isothiocyanate goat antimouse IgG was purchased from BOSTER (Wuhan, China).

## 3. Results

### 3.1. Genomic Sequences of Strain 2022FZ

The whole genomic sequence of MDGPV/2022FZ contained 5,071 nucleotides. The inverted terminal repeats (ITRs) of the 5′ and 3′ terminal regions consisted of 424 nucleotides and were folded to establish hairpin structures. The NS and VP genes consisted of 1,884 and 2,199 nucleotides encoding 627 and 732 aas, respectively. The whole genome sequence of the 2022FZ strain was deposited in GenBank under the accession number OR157985. This generated sequence was compared to a total of 18 strains of waterfowl parvovirus, and the full-length sequence homology of 2022FZ compared to the classical GPV, classical MDPV, and MDGPV strains was 84.7%–85.6%, 89.3%–91.4%, and 97.2%–98.4%, respectively.

### 3.2. Recombination Analysis of Strain MDGPV/2022FZ

**Figure 1:**
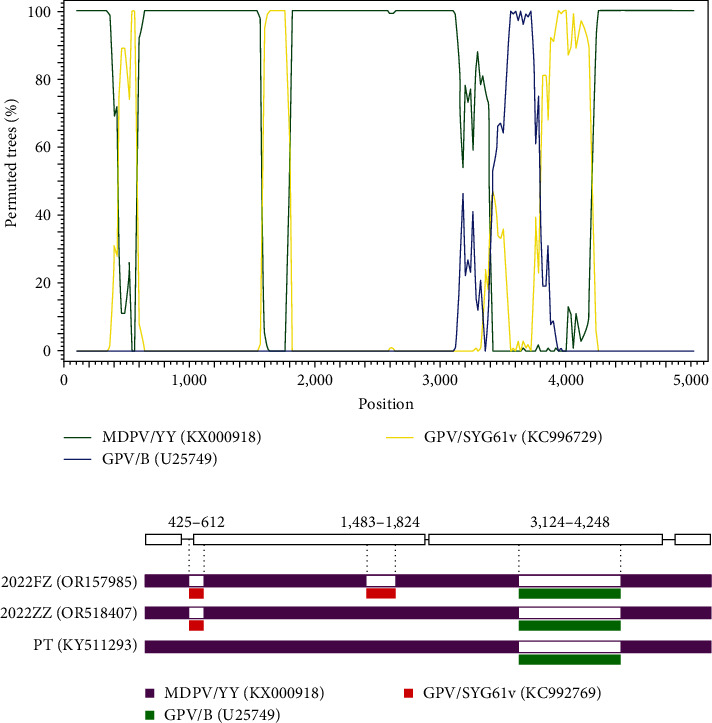
(a) The 2022FZ strain recombination analysis. Recombinations occurred in the VP3 gene, P9 promoter-NS region, and NS2 gene, respectively. In these recombination events, the classical MDPV/YY acted as the major parent, and the classical GPV strain B and attenuated strain SYG61v served as the minor parents. (b) Recombination events occurred gradually replaced, rather than occurring simultaneously.

Recombination analysis revealed that MDGPV/2022FZ underwent three restructuring events in the VP3 gene, P9 promoter-NS gene, and NS2 gene regions, respectively (Figure 1(a)). The three region recombinants in the 2022FZ strain were replaced gradually rather than occurring simultaneously (Figure 1(b)). In the VP3 recombination event, the breakpoint started at nucleotide position 3,124 and ended at nucleotide position 4,248. The classical MDPV/YY acted as the major parent, and the GPV/B served as the minor parent. The MDGPV/2022FZ strain and the previously characterized recombinant strains PT, ZW, NM100, SAAS-SHNH, and GD201911 shared highly consistent recombination in this region.

In the P9 promoter-NS recombination event, the 188-nucleotide recombination region extended from the end of the 5′ ITR region to the 96th nucleotide downstream of the NS gene's initiation codon. In this recombination event, MDPV/YY served as the major parent, and the GPV attenuated vaccine strain SYG61v acted as the minor parent. The previously characterized recombinant strains ZW, NM100, SAAS-SHNH, and GD201911 also experienced this recombination event, but strain MDGPV/PT, which is the earliest recombinant strain, did not experience recombination in this region.

Apart from the two typical restructuring events mentioned above, a novel recombination event was identified in the NS2 gene of MDGPV/2022FZ. The recombinant region contained 342 nucleotides and shared 99.7% homology with the SYG61v strain but 85.7%–86.0% and 86.0%–86.5% homology with the MDGPV and classical MDPV strains, respectively. Here, the 342 nt of the NS2 region (starts at nucleotide position 1,483 and ends at position 1,824) of the 2022FZ strain was replaced by the homologous sequence from strain SYG61v. This recombinant event also occurred with MDPV/YY as the major parent and GPV/SYG61v as the minor parent.

### 3.3. Sequence Comparison of NS2 Gene Recombination Region

**Figure 2:**
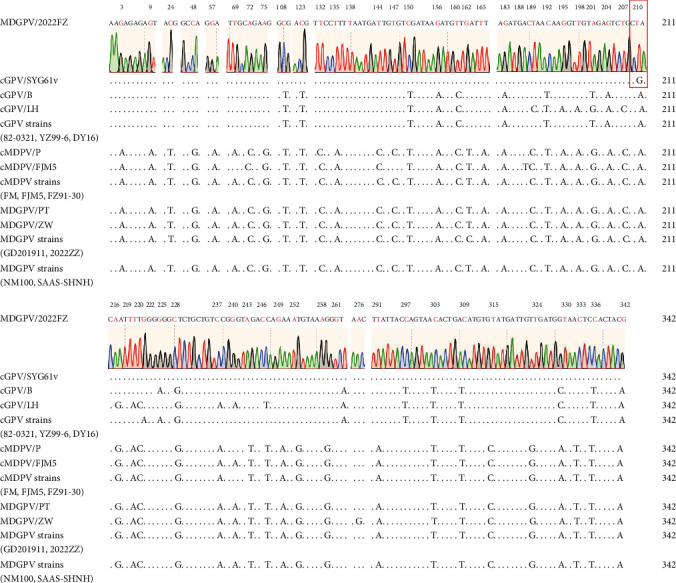
The alignment of the 342 nucleotide recombination region in the NS2 gene of 2022FZ. Dots denote nucleotides identical between 2022FZ and other waterfowl parvovirus strains. The 2022FZ strain shares almost 100% nucleotide homology with SYG61v, with only one nucleotide difference at position 210.

**Figure 3:**
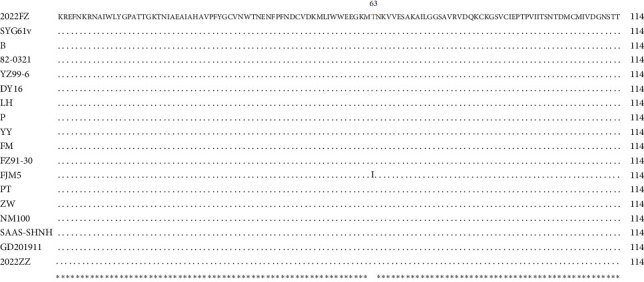
The alignment of the 114 aa recombination region in the NS2 gene of 2022FZ. Dots denote aas identical between 2022FZ and other waterfowl parvovirus strains. The 2022FZ strain shares almost 100% aa homology with SYG61v, except for one mutation from Thr to Ile in the FJM5 strain at position 63.

Compared with the GPV attenuated strain SYG61v, the recombination region in the NS2 gene of 2022FZ only had one nucleotide difference at position G210T and showed obvious differences in nucleotide homology with other MDGPV or MDPV strains (Figure 2). Interestingly, this recombination event resulted in only one aa level change from threonine to isoleucine at aa position 63 in the FJM5 strain (Figure 3).

### 3.4. Homology Analysis of the ITRs Region

**Figure 4:**
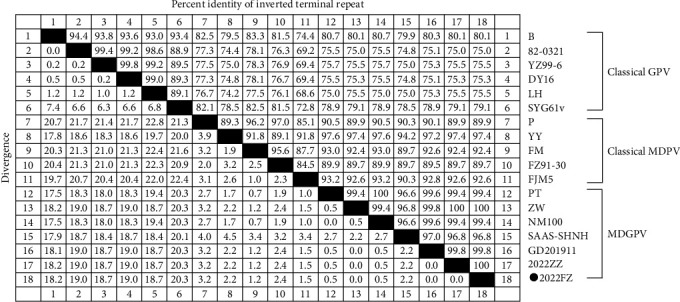
The nucleotide homology of the ITR region of 18 waterfowl parvoviruses.

Among the 18 strains of waterfowl parvovirus, the ITR region of strain 2022FZ had 100.0% homology with ZW, followed by GD201911 (99.8%). The ITR homology between 2022FZ and the classical MDPV strains was 88.8%–97.2%, while homology with the classical GPV virulent strain was only 73.8%–79.4% (Figure 4). These results suggest that the ITR of 2022FZ was derived from the classical MDPV strains.

### 3.5. Sequence Comparisons of the NS and VP Proteins

**Figure 5:**
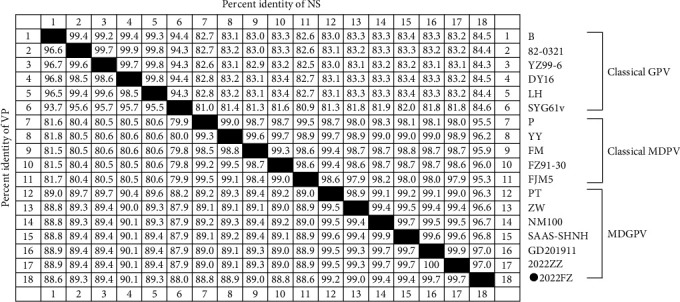
A percent similarity of the NS (lower left) and VP (upper right) genes shared by 18 waterfowl parvovirus strains.

As shown in Figure 5, the nucleotide sequence of the 2022FZ NS gene had a higher nucleotide similarity with MDPV strains (95.3%–96.2%) than that of GPV strains (84.3%–84.6%) and shared 96.3%–97.0% similarity with other MDGPV strains. The VP gene of the 2022FZ strain had 88.0%–90.1% similarity with GPV, 88.6%–89.0% similarity with MDPV, and more than 99.0% nucleotide homology with the MDGPV strains.

**Figure 6:**
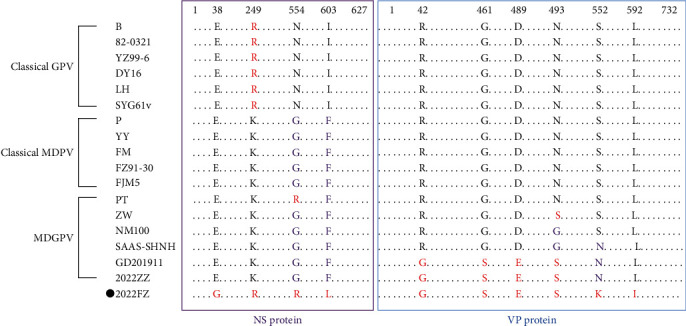
Ten aa mutations were introduced in the NS and VP proteins of the 2022FZ strain of waterfowl parvoviruses.

As shown in Figure 6, the aa sequence alignment showed that 2022FZ strains possessed two characteristic aa mutations in the NS protein at positions 38 (E→G) and 603 (I or F→L), respectively, that are distinct from other waterfowl parvovirus strains. The 249^th^ aa in the NS gene of 2022FZ is a classical GPV site, while the 554th aa was the same as in the PT strain. The VP protein also had two characteristic mutations at position 552 (S or N→K) and 592 (L→I). The aa substitutions at positions 42 (R→G), 461 (G→S), and 489 (D→E) were identical to the GD201911 strain and the 2022ZZ strain, while that of position 493 (N or G→S) was consistent with the ZW strain, the GD201911 strain, and the 2022ZZ strain.

### 3.6. Phylogenetic Analysis of Strain 2022FZ

**Figure 7:**
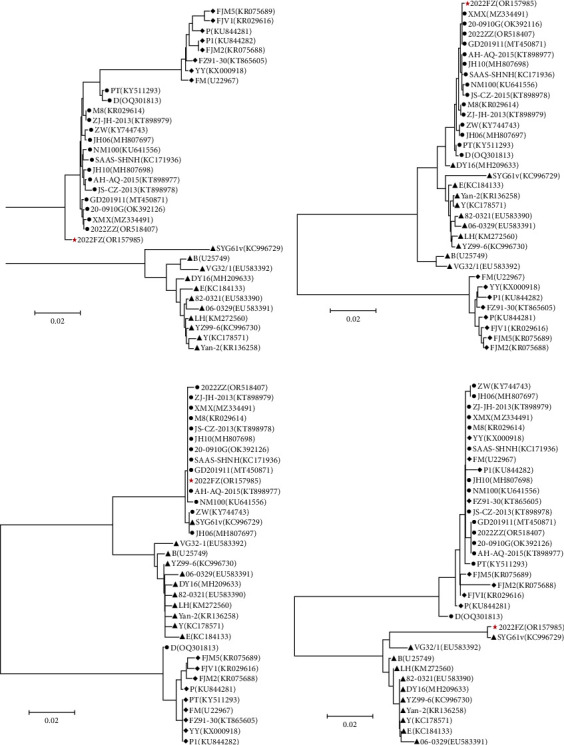
Neighbor-joining phylogenetic trees of whole genomic sequences and recombinant gene regions of the 2022FZ waterfowl parvoviruses. (a) the whole genome, (b) the NS2 recombinant region, (c) the VP3 recombinant region, and (d) the P9 promoter-NS recombinant regions. Branch support was assessed with 1,000 bootstrap replications. The novel three-region recombinant strain, 2022FZ, identified in this study is highlighted as red star. The black triangles represent the classical GPV strains; the black diamonds represent classical MDPV strains; the black circles represent MDGPV strains.

Phylogenetic trees were constructed based on the whole genomic sequence and recombinant domains in the VP3 gene, the P9 promoter-NS region, and the NS2 gene, respectively. As shown in Figure 7(a), waterfowl parvoviruses are divided into two groups, namely GPV and MDPV, based on whole genomic sequences. The phylogenetic tree of the whole genome revealed all recombinant waterfowl parvoviruses except 2022FZ to be tightly clustered in a subclade of the MDPV group (Figure 7(a)). Because of the novel recombinant region in the NS2 gene, strain MDGPV/2022FZ formed a distinct clade in the MDPV group (Figure 7(a)). The phylogenetic trees based on the VP3 (Figure 7(b)) and the P9 promoter-NS recombinant regions (Figure 7(c)) showed that all recombinant waterfowl parvoviruses, including 2022FZ, formed a major genetic lineage in the GPV group. According to the phylogenetic analysis of the NS2 recombinant region, 2022FZ formed a distinct clade in the GPV group (Figure 7(d)). The phylogenetic analysis further confirmed that MDGPV strains are recombinant waterfowl parvoviruses, with MDPV forming the genetic backbone and the recombinant sequences in the P9 promoter-NS, NS2, and VP3 regions being derived from GPVs.

### 3.7. Pathogenicity of Strain 2022FZ

**Figure 8:**
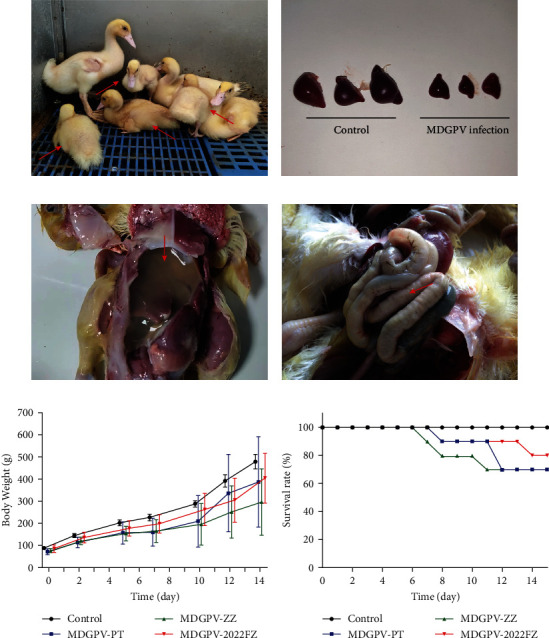
Pathogenicity of MDGPV in 6-day-old Muscovy ducklings: (a) serious weight loss with clinical manifestations (arrow), (b) spleen atrophy, (c) ascites (arrow), (d) enteritis with intestinal embolism (arrow), (e) the weight diversity curve, and (f) the survival rate curve produced over 14 days of infection.

The Muscovy ducklings inoculated with the MDGPV/2022FZ strain exhibited clinical signs similar to those infected with the PT and ZZ strains, including anorexia, locomotor dysfunction (paralysis of legs), weakness (reluctant moving), dwarfism (Figure 8(a)), watery diarrhea, and acute death. The clinical signs appeared 5–6 days after infection, and death occurred 4–9 days after illness, while mortality peaked at 5–7 days after symptom onset. The morbidities of 2022FZ, PT, and ZZ infection were 50%, 70%, and 90%, respectively. The mortalities of 2022FZ, PT, and ZZ infection were 20%, 30%, and 30%, respectively (Figure 8(f)). The degree of incidence and mortality in the MDGPV/2022FZ strain is lower than that of the double recombinant and single recombinant MDGPV strains. Gross lesions were subtle, and spleen atrophy, ascites, and enteritis with intestinal embolism were observed in some birds (Figure 8(b)–8(d)). As shown in Figure 8(e), most of the recovered ducklings showed remarkably poor growth, but the weight loss in the 2022FZ infection group was less serious than those in the PT or ZZ infection groups. All ducklings in the control group remained healthy until the end of the experiment. These results indicate that the pathogenicity of the three-region recombinant MDGPV strain, 2022FZ, in Muscovy ducklings was lower than that of the double recombinant and single recombinant MDGPV strains.

### 3.8. Immunofluorescence Assay

**Figure 9:**
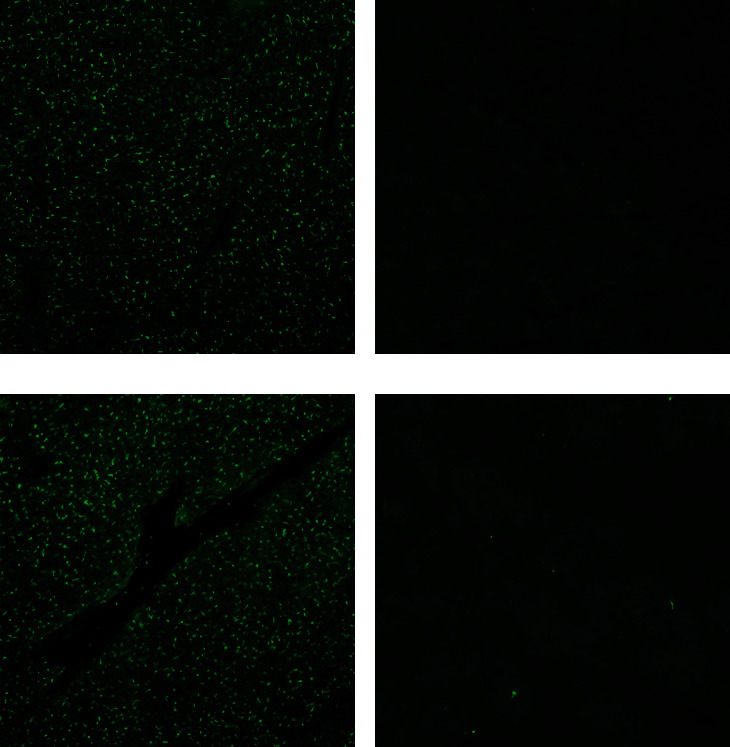
Detection of GPV and MDPV antigens by IFA. GPV and MDPV antigens were detected by specific GPV Mab G-E16 and MDPV Mab M-f21 in heart and liver sections of dead ducks infected with MDGPV/2022FZ. Sections of the heart (a) and liver (c) were stained with GPV Mab G-E16. Sections of the heart (b) and liver (d) were stained with MDPV Mab M-f21.

As shown in Figures 9(a) and 9(c), the heart and liver samples from 2022FZ-infected ducklings were GPV antigen positive, while MDPV antigen was not detected (Figures 9(b) and 9(d)). The heart and liver samples from the control group ducklings were negative for both MDPV and GPV antigens (data not shown).

## 4. Discussion

Evidence of frequent recombination is shown in parvoviruses, for example, *human bocavirus* (HBoV) [21], *porcine parvovirus* (PPV) [22], *canine parvovirus* (CPV) [23], and *Equine parvovirus-hepatitis* (EqPV-H) [24]. It is believed that this genetic recombination was crucial to the emergence of novel parvoviruses. It follows that the frequent recombination and mutation events that waterfowl parvoviruses have experienced in recent decades are not surprising. Short beak and dwarfism syndrome (SBDS) in ducklings is caused by a distinct lineage of GPV, which is formed by genetic divergence and evolution from the classical GPV stain (cGPV) [25]. Waterfowl parvovirus recombination between MDPV strains and GPV strains resulted in the MDGPV strain, which caused Derzy's disease-like symptoms in ducklings, with the typical intestinal embolism. The MDGPV strain appears to be unstable, and it has experienced three generations of evolution after the first recombinant strain MDGPV/PT emerged in 1997. The third-generation strain MDGPV/2022FZ is a result of a three-region recombination between the GPV and MDPV strains.

The whole genome sequence homology between 2022FZ and the MDGPV strains was the highest (97.2%–98.4%) of the 17 waterfowl parvovirus strains evaluated in this investigation, followed by conventional MDPV (89.3%–91.4%) and classical GPV (84.7%–85.6%). The novel recombination event located in the NS2 region (1,483−1,824 nt) was first detected in strain 2022FZ in this study. Additionally, there were some unique aa mutations in the NS and VP proteins, including E38G and I/F603L in the Rep protein and S/N552K and L592I in the VP protein.

All the MDGPV strains use the MDPV virulent strain as the backbone, while the corresponding GPV sequences replace the recombinant regions. Some scholars refer this kind of recombinant waterfowl parvovirus isolates as novel Muscovy duck parvovirus (N-MDPV). Strikingly, our previous studies have shown that the antigen of all the MDGPV strains was more similar to the GPV strains as identified by a virus cross-neutralization test and monoclonal antibody-based LA test [15, 18, 19]. The result in Figure 9 also confirmed MDGPV/2022FZ strain was a GPV-like isolates. The VP3 gene of the initial recombinant strain, MDGPV/PT, has a single region replaced, whereas the VP3 gene of the two- and three-region recombinant strains of MDGPV shares the same recombinant feature. The findings imply that the pathotype and serotype of the MDGPV strains are more akin to those of the GPV strains than of the MDPV strains due to the substitution of a GPV-related sequence in the VP3 region of the strains. Consequently, the GPV vaccine has a better preventive effect against MDGPV infection than the MDPV vaccine. Wang et al. [20] successfully replicated the intestinal embolism in Muscovy ducklings by infecting them with the sole rescued MDGPV strain, rMDPV-ZW. These results showed that the VP3 gene is one of the important determinants of the pathogenicity of waterfowl parvovirus.

The mortality rates of the single recombinant, double recombinant, and triple recombinant MDGPV strains were 20%, 30%, and 30%, respectively. The mortality rate from the MDGPV/2022FZ infection was lower than that from the single or double recombinant strains of MDGPV. The weight loss in the animals infected with MDGPV/2022FZ was consistently less than that of the groups infected with single or double recombinant MDGPV strains. The results showed that MDGPV/2022FZ has a reduced pathogenic effect on Muscovy ducklings. For the first time, our study identified a three-region recombinant MDGPV strain from the Chinese mainland. Further research is needed to verify the effect of these recombinations and mutations in the regulation of viral virulence, replication, and immunity. We also recommend a thorough molecular epidemiological study to ascertain the frequency of this kind.

## Figures and Tables

**Table 1:** Primer pairs designed for the amplification of the whole genomes of strains 2022FZ.

Prime name	Primer sequence	Primer position	Amplification length (bp)
F1	TCATTGGAGGGTTCGTTCGTT	1–21; 5,051–5,071	191
R1	ATGCGCCCGATCAGCCTTGA	172–191; 4,881–4,900	

F2	CGCATGCGCCCGATCTGCCATGAAAAT	187–213	271
R2	GCTTACTGGCTTATATAGGGCAG	435–457	

F3	AATGAGACTCAAGGACAGCAGGAC	381–404	1,144
R3	TCCGTAGAGCCATATGGCATTTCT	1,501–1,524	

F4	AAGACCCAGTTCTGGACATTACTA	1,382–1,405	1,089
R4	ATGCGGCTGCAGTCTCATACCAGT	2,447–2,470	

F5	TGAATGCCTGGAGTGTGAAAGAG	2,232–2,254	1,219
R5	ATGCTCGTCATCCGTAAAGACTTG	3,427–3,450	

F6	GCACGATCAGACGAAGACCATT	3,381–3,402	1,315
R6	CAATGAGACTCAAGGACAGCAGGAC	4,668–4,692	

F7	GGTACCAGATATTTGACTCAGAATC	4,588–4,612	297
R7	GCATGCGCCCGATCTGCCATGAAAAT	4,859–4,884	

**Table 2:** The waterfowl parvoviruses sequences used in the analyses.

Type	Strain	Pathogenicity	Geographic origin	Genome region	Year of isolation	GenBank accession no.
GPV	B	Pathogenic	Europe	Full length	1960s	U25749
	82-0321	Pathogenic	China	Full length	1982	EU583390
	LH	Pathogenic	China	Full length	2012	KM272560
	YZ99-6	Pathogenic	China	Full length	1999	KC996730
	SYG61v	Vaccine	China	Full length	1961	KC996729
	DY16	Pathogenic	China	Full length	2016	MH209633
	Y	Pathogenic	China	Full length	2011	KC178571
	E	Pathogenic	China	Full length	2012	KC184133
	VG32/1	Vaccine	China	Full length	2006	EU583392
	06-0329	Pathogenic	China	Full length	2006	EU583391
	Yan-2	Pathogenic	China	Full length	2013	KR136258

MDPV	FM	Pathogenic	Europe	Full length	1980s	U22967
	P	Pathogenic	China	Full length	1988	KU844281
	P1	Vaccine	China	Full length	n.a.	KU844282
	YY	Pathogenic	China	Full length	2000	KX000918
	FJV1	Pathogenic	China	Full length	2011	KR029616
	FJM2	Pathogenic	China	Full length	2013	KR075689
	FJM5	Pathogenic	China	Full length	2013	KR075688
	FZ91-30	Vaccine	China	Full length	n.a.	KT865605

MDGPV	PT	Pathogenic	China	Full length	1997	KY511293
	D	Vaccine	China	Full length	n.a.	OQ301813
	ZW	Pathogenic	China	Full length	2006	KY744743
	NM100	Pathogenic	China	Full length	2012	KU641556
	SAAS-SHNH	Pathogenic	China	Full length	2012	KC171936
	GD201911	Pathogenic	China	Full length	2019	MT450871
	JH06	Pathogenic	China	Full length	2006	MH807697
	JH10	Pathogenic	China	Full length	2010	MH807698
	M8	Pathogenic	China	Full length	2013	KR029614
	XMX	Pathogenic	China	Full length	2021	MZ334491
	AH-AQ-2015	Pathogenic	China	Full length	2015	KT898977
	JS-CZ-2013	Pathogenic	China	Full length	2013	KT898978
	ZJ-JH-2013	Pathogenic	China	Full length	2013	KT898979
	20-0910G	Pathogenic	China	Full length	2020	OK392126
	2022ZZ	Pathogenic	China	Full length	2022	OR518407
	2022FZ	Pathogenic	China	Full length	2022	OR157985

## Data Availability

The data that support the findings of this study are available on reasonable request from the corresponding author.
